# The Proteolysis of ECM in Intervertebral Disc Degeneration

**DOI:** 10.3390/ijms23031715

**Published:** 2022-02-02

**Authors:** Huaizhen Liang, Rongjing Luo, Gaocai Li, Weifeng Zhang, Yu Song, Cao Yang

**Affiliations:** Department of Orthopaedics, Union Hospital, Tongji Medical College, Huazhong University of Science and Technology, Wuhan 430022, China; 15797891677@163.com (H.L.); luorongjin@hust.edu.cn (R.L.); gaocaili@hust.edu.cn (G.L.); u201510450@hust.edu.cn (W.Z.)

**Keywords:** ECM, IDD, protease

## Abstract

Intervertebral disc (IVD) degeneration (IDD) is a pathological process that commonly occurs throughout the human life span and is a major cause of lower back pain. Better elucidation of the molecular mechanisms involved in disc degeneration could provide a theoretical basis for the development of lumbar disc intervention strategies. In recent years, extracellular matrix (ECM) homeostasis has received much attention due to its relevance to the mechanical properties of IVDs. ECM proteolysis mediated by a variety of proteases is involved in the pathological process of disc degeneration. Here, we discuss in detail the relationship between the IVD as well as the ECM and the role of ECM proteolysis in the degenerative process of the IVD. Targeting ECM proteolysis-associated proteases may be an effective means of intervention in IDD.

## 1. Introduction

Lower back pain is a chronic and widespread musculoskeletal disorder that occurs in approximately more than 85% of people worldwide during their lifetime [1,2]. Disc degeneration has been implicated as a major cause of lower back pain [3,4]. Current clinical treatments for IDD include conservative treatment and surgical intervention. However, these treatment strategies all tend to relief symptoms rather than eliminate the underlying cause [1]. Further exploration of the molecular mechanisms involved in disc degeneration will provide new therapeutic targets to intervene in lower back pain.

The IVD is a complex structure consisting of three parts: the nucleus pulposus (NP) at its core, the annulus fibrosus (AF) surrounding it, and the endplates anchored to the upper and lower sides [5]. Many studies have shown that a variety of genetic or environmental etiologies that damage the nucleus pulposus, annulus fibrosus, or endplate can lead to disc degeneration, with the nucleus pulposus bearing the brunt of the degeneration [6,7,8]. Currently, the nucleus pulposus cells are considered to bear the highest degree of remolding during IDD and maintain the homeostasis of the ECM. In normal IVDs, the anabolism and catabolism of the ECM are in a dynamic balance. When the homeostatic balance of the ECM is disturbed by various stimuli, disc degeneration usually occurs [9]. As disc degeneration progresses, proteoglycans and collagen type II content decrease significantly, while collagen type I content increases significantly, resulting in decreased water absorption, a dysfunctional mechanical microenvironment, and decreased resistance to the loading of the ECM [10,11,12]. Mounting evidence indicate that various proteases play key roles in ECM proteolysis in disc degeneration. Abnormal expression of diverse protease in degenerated disc tissue and its role in ECM proteolysis have been reported. In this review, we systematically describe the role of diverse proteases in IDD. In addition, recent advances in the treatment of disc degeneration by maintaining ECM homeostasis are detailed in this article. A better understanding of the precise mechanisms by which proteases are associated with disc degeneration may provide guidance for the development of therapeutic strategies for disc degeneration

## 2. ECM in IVD

The ECM is a non-cellular structure composed of about three hundred proteins [13]. ECM is present in the extracellular environment of all tissues and is involved in numerous cellular processes [14]. The major ECM components of the IVD include collagens, proteoglycan, and non-collagenous proteins [15]. Fine-tuned ECM dynamics are essential for the normal physiological functioning of the IVD [16]. NP cells are dispersed in a complex network of interwoven polysaccharides and collagen, which continuously interact with the surrounding ECM in a bidirectional manner to maintain tissue homeostasis [15].

### 2.1. Collagens

Collagen is a key component of the ECM and provides mechanical support for the disc cells. By acting on cell membrane receptors, collagen regulates a variety of cellular phenotypes including migration, proliferation, and differentiation [17]. Collagens I and II constitute the bulk of fibrillar matrix of the IVD, with other collagen subtypes including III, V, VI, IX, XI, XII, and XIV [18,19]. As the IVD degenerates, there is a shift from collagen type II to collagen type I [10]. In addition, the distribution of collagens changes, with more collagen II accumulating on the outer annulus and type I collagen accumulating in the nucleus pulposus and inner annulus [20]. 

### 2.2. Proteoglycans

Proteoglycans are macromolecules composed of core proteins and glycosaminoglycan (GAG) side chains. Aggrecan, the most abundant proteoglycan in IVD, is responsible for much of the water-attracting properties due to its high fixed charge density [10]. As age and degeneration progress, aggrecan is cleaved into non-aggregating fractions, leading to reduced hydration of the IVD [15]. Decorin is a member of small leucine-rich proteoglycans (SLRPs), which are widely expressed in connective tissue [21]. Decorin binds to a variety of ECM proteins such as collagen type I, II, III, and VI, which contribute to ECM assembly [22,23]. In addition, decorin binds to various signaling factors to regulate cell proliferation and differentiation and ECM turnover [24,25]. Decorin has been shown to be highly expressed in degenerating discs and may be involved in tissue repair [21]. Versican is a large extracellular proteoglycan that plays important roles in cell division, adhesion, migration, differentiation, and ECM turnover [26,27]. Previous studies have shown that versican is more highly expressed in IVD tissue than in articular cartilage and has a higher abundance in the nucleus pulposus [28]. In addition, in vitro studies have shown that versican exerts a protective effect by promoting the proliferation and adhesion of NP cells [29].

### 2.3. Non-Collagenous Proteins

Elastin is cross-linked by the soluble precursor tropoelastin [30]. Mature Elastin is present in various soft tissue ECM and is the most hydrolytically resistant ECM macromolecule [31]. Elastin constitutes approximately 10% of the IVD matrix and its abundance is higher in the AF compared to the NP [32,33]. Elastin, together with collagen, is thought to play an important role in IVD biomechanics [34,35]. Fibronectin, a ubiquitous secretory glycoprotein, plays a key role in the adhesion of many cell types by interacting with cell surface specific receptors and ECM components [36]. In addition, Anderson et al. showed that all five Fibronectin splice variants are present in IVD tissues [37]. Laminin is a heterotrimeric complex consisting of three polypeptide chains (α, β, and γ) [38]. At least 15 Laminin isoforms are formed by the combination of three different peptide chains, and multiple Laminin isoforms and their receptors are expressed in IVD tissues [38]. In addition, integrin-mediated interaction with Laminin regulates the phenotype of NP cells [39].

## 3. ECM Dysregulation in IDD

Mounting evidence has shown that various genetic and environmental factors, such as reduction in nutrition, overloading biomechanical stress, diabetes mellitus, smoking and infection, can damage NP cells and, thus, cause the dysregulation of ECM homeostasis [6,7,8]. 

First, the process of disc degeneration is accompanied by the progressive loss of notochord-like NP cells and their transformation into chondrocyte-like NP cells, partly attributed to non-physiological loading, inflammation, metabolic dysregulation, and hypoxia [40,41,42,43]. Notochord-like NP cells are essential for ECM homeostasis, and the loss or phenotypic alteration of notochord-like NP cells can lead to ECM dysregulation. Chondrocyte-like NP cells exhibit a different expression profile of ECM components compared to notochord-like NP cells. Chondrocyte-like NP cells tend to express collagen type I, which is capable of forming stronger fibers, accompanied by a reduced amount of more absorbent proteoglycans and collagen type II [44,45]. In addition, the shift from anabolic to catabolic metabolism occurs in IDD. A variety of cytokines expressed by degenerating nucleus pulposus cells upregulate the expression of proteases involved in the remodeling of the ECM [46,47,48,49]. Altogether, there is a decrease in proteoglycans and collagen type II and an increase in collagen type I during the degeneration process. 

Second, changes in the ECM in the process of IDD impact the behavior of NP cells. The degenerative process of the IVD is accompanied by an increase in fibrotic-like collagen type I and a decrease in water-attracting proteoglycans and collagen type II [10,11,12]. The altered ECM composition increases its stiffness and regulates the phenotype and function of NP cells [50]. Available evidence indicates that a stiff substrate induces phenotypic changes including proliferation, apoptosis, and senescence in nucleus pulposus cells and is closely associated with disc degeneration [51]. In addition, ECM remodeling alters the mechanical microenvironment of the IVD. Inappropriate forces imposed on the nucleus pulposus, such as compression, stretch, and shear, impair the functioning of the nucleus pulposus cells [52,53,54,55]. Available evidence suggests that the response of nucleus pulposus cells to sustained mechanical stimuli is closely related to disc degeneration [16,56]. Furthermore, alterations in ECM composition modulate NP cell behavior by directly affecting cell–ECM interactions without altering the mechanical environment [39,57]. Dysregulation of ECM homeostasis is closely related to the process of disc degeneration, and proteolysis of the ECM occurs at the beginning of this process. A better understanding of the role played by ECM proteolysis in the degenerative process of the IVD will contribute to the development of new therapeutics.

## 4. The Proteolysis of ECM in IVD Pathogenesis

### 4.1. Matrix Metalloproteinases (MMPs)

MMPs, a class of Zn+-dependent endopeptidases, were first described in 1962 and are mainly responsible for the degradation of the ECM. To date, a total of 23 human MMPs have been identified [58]. Under normal human physiological conditions, MMPs are in a low expression state. Injury remodeling processes and an inflammatory environment increase their expression [59]. MMPs are first secreted as zymogens and are activated in the extracellular space by the hydrolytic cleavage of inactive structural domains or oxidative modification of thiol groups [60]. Importantly, MMPs are involved in a wide range of biological processes and are closely associated with disease development [61,62,63].

MMP expression is low in normal IVD tissue but increased in degenerative IVD tissue [20]. Studies using human IVD specimens show that MMPs increase gradually with an increasing grade of IVD degeneration [64]. In addition, highly expressed MMPs have been described in multiple animal models of degenerative discs [65,66,67,68]. Collectively, this evidence indicates that the level of MMPs expression is positively correlated with the grade of IVD degeneration. It also has been demonstrated that highly expressed MMPs are involved in IDD pathogenesis. Under pressure overload conditions, NP cells exhibit abnormal MMPs expression and corresponding ECM degeneration. Omlor et al. demonstrated a progressive elevation of MMP-13 with sustained applied pressure, which resulted in enhanced catabolism of the ECM [69]. Using a rat tail static stress model, Yurube found an imbalance in the expression of MMPs and anti-catabolic proteins [70]. Similarly, Yan et al. showed that compression loading altered the expression of multiple MMPs [71]. In addition, the inflammatory microenvironment demonstrated a significant effect on the expression of MMPs. Zhang et al. found disc degeneration accompanied by elevated expression of inflammatory cytokines and catabolic enzymes in a goat disc degeneration model [72]. It has been proven that inflammatory cytokines indirectly induce the hydrolytic activity of MMP-2 by elevating the expression of MT-MMP [73]. In addition, much evidence suggests that oxidative stress is also closely associated with over-activated MMPs. Reactive oxygen species (ROS) are involved in the transduction of multiple intracellular signaling pathways, but excessive ROS production can impair cellular function. In an earlier study, Nasto and colleagues showed that mitochondria-derived ROS degenerate in myeloid cells by upregulating the expression of MMP-1 and MMP-3 [74]. Dimozi showed that exogenous ROS addition can also induce the expression of ECM enzymes including MMP-1, MMP-3, and MMP-9 in hydrogen peroxide-treated NP cells [75]. There is increasing evidence that high glucose is closely associated with excessive ROS production, and Cheng et al. showed that oxidative stress impairs ECM metabolic homeostasis through the activation of p38/MAPK in high glucose-induced degenerating NP cells [76]. In a recent study, Liu et al. showed that oxidative stress resulted in decreased expression of the antioxidant protein FOXOs and upregulated expression of MMP-13 [77]. Furthermore, a range of therapies targeting MMPs has been explored to treat disc degeneration. Cheng et al. used hydrogel delivery of ferulic acid targeting MMP-3 to improve ECM homeostasis for the treatment of IDD [78]. Using a bioresponsive hydrogel loaded with miR-29a, Feng et al. proposed a strategy for targeting MMP-2 to treat disc degeneration [79]. Further exploration of the precise mechanisms of MMPs is needed to develop promising therapies for IDD.

### 4.2. A Disintegrin and Metalloprotease with Thrombospondin Motifs (ADAMTSs)

ADAMTS is a secreted protease of the metalloproteinase family that contains a thrombospondin structural domain that allows it to bind to ECM components, which can further lead to ECM proteolysis [80,81]. ADAMTS can be further classified into four categories based on their function and preferential ECM substrates. The hyalectanases (ADAMTS-1, ADAMTS-4, ADAMTS-5, ADAMTS-8, ADAMTS-9, ADAMTS-15, and ADAMTS-20) mainly target proteoglycans, while pro-collagen N-propeptidases (ADAMTS-2, ADAMTS-3, and ADAMTS-14) mainly target collagens I, II, and III. ADAMTS-13 is involved in the cleavage of the von Willebrand factor, which contributes to the pathophysiology of coagulation and thrombotic thrombocytopenic purpura. The function of the remaining ADAMTS (ADAMTS-6, ADAMTS-7, ADAMTS-10, ADAMTS-12, ADAMTS-16, ADAMTS-17, ADAMTS-18, and ADAMTS-19) is unclear.

Degenerative aggrecan is an important biochemical feature of IDD. The hyalectanases are responsible for aggrecan degradation in disc degeneration [82]. Pockert et al. demonstrated that ADAMTS-1, 4, 5, 9, and 15 accumulate in degenerating disc tissue and may contribute to the alteration of the ECM during disc degeneration [83]. Wang et al. found that inflammatory cytokines regulate ADAMTS-5 expression through SDC4, which is involved in the pathophysiology of disc degeneration [84]. Using a rat tail static compression model, high expression of ADAMTS-4 and ADAMTS-5 was observed [70]. In addition, the direct degradation effect of ADAMTS-4 on the ECM of the nucleus pulposus was assessed. It was shown that intradiscal injection with ADAMTS-4 impaired cell activity and collagen content [85]. It also has been demonstrated that sIL-13Rα2-Fc is able to rescue ECM degradation by targeting ADAMTS-8 [86]. Collectively, these studies indicated that the hyalectanases play key roles in the pathophysiology of disc degeneration. In addition to hyalectanases, other ADAMTSs have been reported to be associated with disc degenerative processes. For example, McCann et al. found that TNF-α and ADAMTS-7 were highly expressed in IL-21-treated NP cells, and further studies showed that inflammatory factor-induced expression of multiple catabolic enzymes was ADAMTS-7-mediated [87,88]. In addition, Yu et al. found elevated expression of ADAMTS-7 and ADAMTS-12 in degenerated disc tissue accompanied by degradation of the cartilage oligomeric matrix protein [89]. Further investigation of the role of ADAMTSs in IDD etiology is critical for IDD intervention.

### 4.3. Cathepsins Proteases

Cathepsins belong to the peptidases family, which are primarily involved in the endosomes and lysosomes for protein degradation [61]. There is increasing evidence indicating that cathepsins are also expressed in the extracellular space and are involved in the degradation of the ECM [90]. Cathepsins are composed of different protease families: cysteine, serine, and aspartyl proteases. Cathepsins play an important role in a variety of diseases, such as bone and joint degenerative diseases, and are receiving widespread attention [61].

Dando et al. described a rabbit IVD model in which intradisc injection of Cathepsins B resulted in proteoglycan breakdown and histological and imaging alterations of the IVD [62]. Further studies demonstrated that Cathepsins B was highly expressed in degenerating discs [63]. Ariga et al. found that cathepsins D, K, and L accumulate at the site of degenerated discs and that this specific localization may be associated with ECM disorders during disc degeneration [91]. In addition, Gruber et al. found that cathepsins K expression was significantly elevated in degenerated discs and positively correlated with receptor activator of the NF-κB ligand [92]. In a zebrafish model, the impact of high bone mineral density induced by mutant cathepsins K on IVDs was examined [93]. It was found that high bone mineral density was significantly associated with disc degeneration. Additionally, Konttinen et al. demonstrated a significant increase in cathepsins K-positive cells in degenerating IVDs [94]. These studies suggest that cathepsins play key roles in IDD.

### 4.4. Other Proteases

High temperature requirement A1 (HTRA1) belongs to the serine protease family and is capable of degrading a variety of ECM components. Tiaden et al. found that HTRA1 expression was elevated in degenerating discs, and further studies showed that HTRA1 could upregulate various catabolic enzymes [95]. Hepsin (HPN) is also known as transmembrane Serine Protease 1 (TMPRSS1), whose overexpression leads to degradation of the IVD ECM [96]. Plasmin is involved in the activation of several MMPs [97]. Salo et al. showed that plasmin was present in degenerating disc tissue with high expression of multiple catabolic enzymes, suggesting that plasmin may be associated with anabolic imbalance during disc degeneration. In addition, Campos et al. demonstrated that heparanase isoforms (HPSE1 and HPSE2) are highly expressed in degenerated disc tissues [98].

## 5. Therapeutic Targeting of Proteases for ID

Current clinical treatments for IDD are aimed at relieving symptoms rather than directly targeting disc degeneration. Given the critical role of ECM proteolysis driven by proteases in the process of disc degeneration, targeting proteases in disc degeneration is an alternative option. Here we review recent advances in modulating ECM homeostasis for the treatment of IDD (Table 1). 

### 5.1. Anti-Inflammation

Inflammatory stimulation is one of the most important etiologies of disc degeneration, emphasizing its ability to upregulate multiple proteases. Anti-inflammatory therapy demonstrates the potential for treating disc degeneration. The NF-κB pathway has been reported to mediate the transcriptional activation of multiple hydrolases in response to inflammatory stimuli. In cytokine-induced inflammatory models, multiple bioactive compounds were shown to downregulate the expression of multiple proteases by inhibiting NF-κB, which in turn blocked the ECM destruction process [99,100,101]. In addition, Li et al. found that Crocin and Sesamin exerted anti-inflammatory effects by inhibiting the activation of the JNK pathway and slowed down the degradation of the degenerating IVD ECM [102,103]. It has also been reported that Platelet-Rich Plasma (PRP) to treat disc degeneration, and the results showed that PRP treatment promoted the production of major components of the ECM and inhibited the expression of MMP-1/3 [104,105]. These studies suggest that inhibition of inflammation represents a new class of treatment for disc degeneration (Table 1).

### 5.2. Anti-Oxidation

In recent years, there has been increasing evidence that oxidative stress is closely related to the imbalance of ECM homeostasis in degenerated discs. Using a rat model of IDD, Qin et al. found that Danshen effectively attenuated the disc degeneration process by targeting the oxidative reaction [106]. Wang et al. demonstrated that Genistein (GES) treatment rescued the expression levels of aggrecan and type II collagen in TBHP-treated NP cells. The role of GES in restoring ECM homeostasis was further demonstrated to be achieved through Nrf2-mediated anti-proteases [107]. 1,4-dihydropyridine (DHP), a specific sirt1 agonist, was shown to be a potential therapeutic agent for IDD due to its antioxidant effect. The administration of DHP significantly alleviated the degeneration of the ECM by reducing the expression of MMP-3 and ADAMTS-5 in degenerating NP cells [108]. Aspirin is widely used as an anti-inflammatory agent for the treatment of low back pain. Liu et al. found that aspirin exerts antioxidant and anti-inflammatory effects in an AMPK-dependent manner. Aspirin treatment protected NP cells from ECM degradation by inhibiting the expression of multiple hydrolases, including MMP-3/13 and ADAMTS-5. Furthermore, aspirin intervertebral injections significantly hindered the degenerative process of the IVD in a rat model [126]. In addition, melatonin and N-acetyl cysteine (NAC) were reported to maintain ECM homeostasis by regulating redox homeostasis [127,128]. These studies suggest that antioxidant drugs may be a promising therapeutic strategy for disc degeneration, emphasizing their modulation of ECM homeostasis (Table 1). 

### 5.3. Stem Cells Therapy

Recently, many studies have focused on stem cell therapy for disc degeneration by the injection or transplantation of stem cells, which aim to reconstitute the ECM of the IVD [129]. Previous studies have reported that the co-incubation of nucleus pulposus cells with stem cells can induce stem cell differentiation, leading to regenerative repair of degenerated discs [110]. Further, Yang and his colleagues used bone marrow derived mesenchymal stem cells (BMSCs) injections to treat disc degeneration. They showed that BMSCs transplantation alleviated disc degeneration by stimulating endogenous ECM regeneration in addition to autonomous differentiation [109]. Yi et al. used human tissue inhibitor of metalloproteinase 1 (TIMP-1) overexpression modified BMSCs to treat degenerated discs by directly targeting the ECM anabolic catabolic homeostasis [111]. In addition, Wang et al. found that BMSCs promoted ECM regeneration in degenerated NP cells via the miR-101-3p/EIF4G2 axis [130]. However, stem cell injections or transplants alone often fail to achieve the desired effect due to their lack of protection and support by the ECM. In a recent study, Feng et al. used an injectable microsphere scaffold for the delivery of stem cells, which provided a suitable environment for stem cell implantation, proliferation, and differentiation, and significantly improved the efficacy of stem cell therapy for degenerated discs [131]. In a similar study, Ukeba et al. used ultra-purified alginate gels loaded with BMSCs to treat degenerated discs, which significantly promoted ECM synthesis with significantly higher efficacy than BMSCs injection alone [132]. In addition, Frapin et al. treated disc degeneration by modulating the distribution of endogenous disc stem/progenitor cells. Chemokine CCL-5 was delivered to the nucleus pulposus via pullulan microbeads (PMBs) to recruit disc stem/progenitor cells, which reversed the phenotype of degenerating NP cells [112]. Collectively, substantial evidence suggests that stem cell injection or transplantation is an effective treatment for targeting the degenerating ECM, but further studies are needed to assess their safety and efficacy (Table 1). 

### 5.4. Metabolic Modulation

Metabolic diseases such as the hyperglycemic microenvironment produced by diabetes mellitus and estrogen deficiency-induced bone loss have been shown to be closely associated with disc degeneration [77,133,134]. Marein, a plant active ingredient with antidiabetic effects, was shown to alleviate degeneration of the ECM of the degenerating NP cells due to high glucose exposure [113]. In addition, anti-inflammatory combined with anti-advanced-glycation-end-products (AGEs) treatment significantly improved disc degeneration in a mouse model of diabetes mellitus [114]. Using an ovariectomized rat model, Song et al. found that alendronate (ALN) treatment significantly retarded the progression of disc degeneration. ALN not only reduced the expression of multiple hydrolases and type I collagen, but also promoted the expression of aggrecan and type II collagen, and the underlying mechanism may be related to the maintenance of disc structural integrity [115,116,117]. In conclusion, these data suggest that the imbalance in metabolic homeostasis may be a potential target for the degeneration of the IVD ECM (Table 1).

### 5.5. Biomaterials

In recent years, novel bioactive materials based on bioengineering have been widely used to treat disc degeneration. Cheng et al. constructed an injectable thermosensitive chitosan–gelatin–glycerol phosphate (C/G/GP) hydrogel for the treatment of disc degeneration by the controlled release of ferredoxin acid. This study demonstrated that C/G/GP gels are well adapted to ferulic acid and achieve controlled release through their thermosensitive properties to target oxidative stress-induced ECM degeneration [78,118]. Using a bioresponsive hydrogel loaded with miR-29a, Feng et al. proposed a strategy for targeting MMP-2 to treat disc degeneration. In this study, the investigators exploited the hyperactivation of proteases in degenerating discs to prepare a hydrolase-responsive miRNA delivery system to regulate ECM homeostasis [79]. Moreover, Larrañaga et al. prepared tannic acid (TA)-functionalized polymer capsules to target MMP-3 and ADAMTS-5 for the treatment of IVDs. The antioxidant functionalized polymer capsules prepared in this study represent the application of polymer capsules for the treatment of oxidative stress-induced ECM degeneration [119]. More recently, a controllable release hydrogel was prepared for the delivery of aspirin. This study demonstrated that controlled release gels carrying aspirin significantly alleviated the ECM degeneration of the IVDs and had superior mechanical properties [120]. Additional efforts to treat degenerating discs include injectable microspheres loaded with tumor necrosis factor (TNF) receptor type II. This antagonist microsphere significantly slows disc degeneration by targeting disorders of ECM metabolism [121]. Similarly, a GelMA microsphere coupled with the active peptide APETx2 was shown to modulate local inflammation overactivation in the degenerated IVD. This study suggests that modulation of the inflammatory microenvironment is a potential means of targeting ECM degradation [122]. In recent studies, caveolae associated protein 2 (Cavin-2) modified engineered extracellular vesicles were used to treat disc degeneration. Cavin-2 modification significantly increased the extracellular vesicle uptake efficacy in degenerating NP cells compared to stem cell-derived extracellular vesicles alone [135]. Collectively, these studies demonstrate the great potential of bioactive materials in targeting IVD ECM degeneration, but more studies are needed to evaluate their long-term safety and efficacy (Table 1).

### 5.6. Gene Therapy

Recently, gene therapy has received increasing attention. There is evidence that gene therapy has the potential to treat IDD [136,137]. Given the important role of multiple proteases in the imbalance of ECM metabolism, targeting proteases through gene therapy may provide a new strategy for the treatment of IDD. Using a rabbit model of IDD, Leckie et al. showed that AAV2-TIMP1 effectively alleviated disc degeneration by increasing collagen type II levels [123]. Similarly, another study showed that TIMP1 co-transduction with transforming growth factor (TGF-β3) and connective tissue growth factor (CTGF) promoted the synthesis of ECM in degenerated discs [124]. In addition, ADAMTS-5 siRNA was shown to retard disc degeneration by directly targeting the protease ADAMTS-5 [125]. Thus, transduction of endogenous proteases inhibitors and direct targeting of proteases have important potential applications in the treatment of IDD (Table 1).

Despite the great progress made in exploring IDD therapeutic strategies, many challenges remain in its translational future. The avascularity of the nucleus pulposus results in a lack of nutrient supply to the core region of the disc. The poor microenvironment of the IVD is a great obstacle to the treatment of IDD [133,134]. Proteases activation is thought to be a driver of ECM degradation in the disc; however, IDD treatment strategies targeting proteases have also failed to meet expectations due to transient effects [138]. Overall, further studies in developing therapeutic intervention for IDD should take these factors into account.

## 6. Conclusions

**Figure 1:**
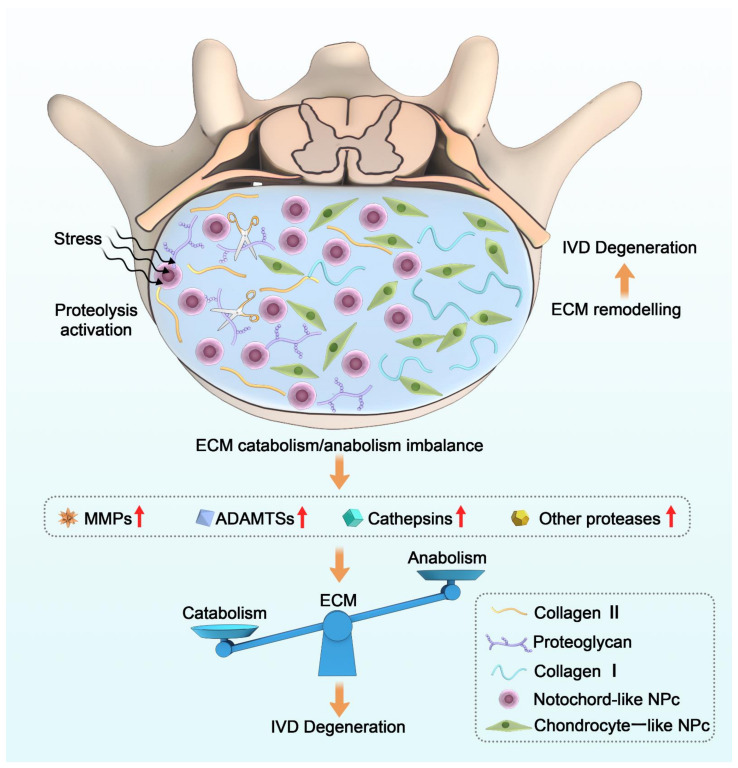
General mechanisms of the pathological process of IDD and the role of proteases in the imbalance of ECM metabolism. Proteolysis activation leads to an imbalance in ECM homeostasis when the IVD is subjected to various stimuli, in which proteases play a key role.

The imbalance of ECM homeostasis is closely related to the pathological process of disc degeneration. ECM proteolysis, mediated by multiple hydrolases in the extracellular space, plays an initiating role in this process (Figure 1). Here, we describe in detail the role of the major proteases in disc degeneration and list strategies for targeting the ECM to treat degenerative discs. 

Given the paucity of therapeutic options for disc degeneration and the critical role of proteases in this regard, targeting one or more proteases demonstrates potential for treating disc degeneration. However, the regulation and precise mechanisms of key proteases in disc degeneration are still largely unknown. Further research is needed that focuses on the molecular mechanisms of proteases regulation and to develop effective treatments for disc degeneration that target proteases. 

## Figures and Tables

**Table 1:** Recent advances in modulating ECM homeostasis for the treatment of IDD.

Strategies	Interventions	Effects	Reference(s)
Anti-inflammation	Cardamonin, Salubrinal, Suramin	Downregulate multiple proteases by inhibiting NF-κB	[99,100,101]
	Crocin, Sesamin	Downregulate multiple proteases by inhibiting JNK	[102,103]
	PRP	Downregulate multiple MMPs and increase levels of several beneficial growth factors	[104,105]
Anti-oxidation	Danshen	Downregulate MMP-3	[106]
	Genistein	Downregulate MMP-13 via Nrf2-mediated antioxidant system	[107]
	DHP	Downregulate MMP-3 and ADAMTS-5 via activating sirtuin-1	[108]
Stem cells therapy	Stem cell implantation	Induce differentiation of stem cells into nucleus pulposus cells and stimulate endogenous ECM regeneration	[109,110]
	TIMP-1 overexpression modified BMSCs	Modulate ECM anabolic catabolic homeostasis	[111]
	Chemokine CCL-5	Recruit disc stem/progenitor cells to nucleus pulposus	[112]
Metabolic modulation	Marein	Downregulate MMP-3 and MMP-13	[113]
	Pyridoxamine	Downregulate MMP-13 and ADAMTS-5 by antagonizing AGE	[114]
	ALN	Downregulate MMP-1, MMP-3 and MMP-13	[115,116,117]
Biomaterials	FA-G/C/GP hydrogel	Downregulate MMP-3 and upregulate aggrecan and type II collagen	[78,118]
	TA-functionalized polymer capsules	Downregulate MMP-3 and ADAMTS-5	[119]
	Aspirin controllable release hydrogel	Downregulate MMP-3/13 and ADAMTS-4/5	[120]
	Injectable microspheres load with TNFRII or APETx2	Downregulate MMP-3 and ADAMTS-5 via modulate local inflammation microenvironment	[121,122]
Gene therapy	AAV2-TIMP1	promote synthesis of type II collagen	[123]
	TGF-β3, CTGF and TIMP1 co-transduction	Promote synthesis of aggrecan and type II collagen	[124]
	ADAMTS-5 siRNA	Downregulate ADAMTS-5	[125]

PRP: Platelet-Rich Plasma, DHP: 1,4-dihydropyridine, TIMP-1: tissue inhibitor of metalloproteinase 1, CCL-5: C-C motif chemokine ligand 5, ALN: alendronate, TNFRII: tumor necrosis factor receptor type II. TGF-β3: transforming growth factor, CTGF: connective tissue growth factor, ADAMTS: A disintegrin and metalloprotease with thrombospondin motifs, MMP: Matrix metalloproteinase.

## Data Availability

Not applicable.
